# PeaTAR1B: Characterization of a Second Type 1 Tyramine Receptor of the American Cockroach, *Periplaneta americana*

**DOI:** 10.3390/ijms18112279

**Published:** 2017-10-30

**Authors:** Wolfgang Blenau, Sabine Balfanz, Arnd Baumann

**Affiliations:** 1 Faculty of Biology, Phillips University of Marburg, 35037 Marburg, Germany; wolfgang.blenau@biologie.uni-marburg.de; 2 Institute of Complex Systems—Cellular Biophysics (ICS-4), Forschungszentrum Jülich, 52428 Jülich, Germany; s.balfanz@fz-juelich.de

**Keywords:** cAMP, biogenic amines, Ca^2+^ fluorimetry, cell-based assay, cellular signaling, G protein-coupled receptor, insect, octopamine, second messenger

## Abstract

The catecholamines norepinephrine and epinephrine regulate important physiological functions in vertebrates. In insects; these neuroactive substances are functionally replaced by the phenolamines octopamine and tyramine. Phenolamines activate specific guanine nucleotide-binding (G) protein-coupled receptors (GPCRs). Type 1 tyramine receptors are better activated by tyramine than by octopamine. In contrast; type 2 tyramine receptors are almost exclusively activated by tyramine. Functionally; activation of type 1 tyramine receptors leads to a decrease in the intracellular concentration of cAMP ([cAMP]_i_) whereas type 2 tyramine receptors can mediate Ca^2+^ signals or both Ca^2+^ signals and effects on [cAMP]_i_. Here; we report that the American cockroach (*Periplaneta americana*) expresses a second type 1 tyramine receptor (PeaTAR1B) in addition to PeaTAR1A (previously called PeaTYR1). When heterologously expressed in flpTM cells; activation of PeaTAR1B by tyramine leads to a concentration-dependent decrease in [cAMP]_i_. Its activity can be blocked by a series of established antagonists. The functional characterization of two type 1 tyramine receptors from *P. americana*; PeaTAR1A and PeaTAR1B; which respond to tyramine by changing cAMP levels; is a major step towards understanding the actions of tyramine in cockroach physiology and behavior; particularly in comparison to the effects of octopamine.

## 1. Introduction

The American cockroach, *Periplaneta americana*, is an established model organism in neurobiological, physiological, and toxicological studies [1,2,3]. Because of its generous size and simple animal maintenance, *P. americana* is well suited for studying the morphology, physiology, and pharmacology of the insect nervous system. Such research has led to a better understanding of the neural basis of escape behavior [4,5], circadian rhythmicity [6,7], and neuropeptide distribution and action [8,9,10]. In addition, various learning paradigms have been established for *P. americana* during the last few years [11,12,13,14,15]. Because of the easy accessibility of intact mini-organs for opto- and electro-physiological recordings, *P. americana* has also been used to study epithelial physiology and to investigate, for example, stimulus-secretion coupling in exocrine glands [16,17,18,19,20].

In residential areas, *P. americana* can infest any type of buildings and is frequently found in basements and sewers. They feed on human and pet food and can leave an offensive odor. They can passively transport pathogenic bacteria, such as *Salmonella*, on their body surfaces, particularly in environments such as hospitals [21,22]. When these bacteria are later deposited on foodstuff, they can cause food infections or poisoning of consumers. In addition, house dust containing cockroach feces and body parts can trigger allergic reactions and asthma in certain individuals [23,24,25]. Thus, *P. americana* must be considered as an insect pest of significant public health importance and cockroach populations may be controlled using insecticides.

Certain classes of insecticides, such as plant essential oils [26,27,28,29] and formamidines [30,31,32,33,34,35], exert their bioactivity primarily by interacting with tyramine and/or octopamine receptors [36,37,38,39]. The phenolamines tyramine and octopamine belong to a group of messenger substances commonly known as biogenic amines. Octopamine and tyramine act as neurotransmitters, neuromodulators and/or neurohormones in insects and other protostomes and have a significant involvement in the regulation of physiology and behavior of these animals [39,40,41,42,43,44,45]. Both phenolamines exert diverse effects by binding to and activating membrane receptors that belong to the large family of G protein-coupled receptors (GPCRs). For each phenolamine, multiple receptor subtypes exist that couple to various intracellular signaling pathways in a receptor specific manner [39,41,43,46,47,48,49]. Octopamine receptors either evoke Ca^2+^ release from intracellular stores (α-adrenergic like octopamine receptors, e.g., [50,51,52,53,54,55]) or activate adenylyl cyclases, thereby increasing intracellular cAMP concentrations ([cAMP]_i_); β-adrenergic-like octopamine receptors, e.g., [52,54,56,57,58]). Type 1 tyramine receptors inhibit adenylyl cyclase activity and thus lead to a reduction in [cAMP]_i_ (e.g., [33,59,60,61,62,63,64]). More recently, members of a second class of tyramine receptors (type 2 tyramine receptors) have been characterized in *Drosophila melanogaster* [65,66], the silkworm *Bombyx mori* [67], and the honeybee *Apis mellifera* [68]. The *D. melanogaster* type 2 tyramine receptor (DmTAR2) seems to bind exclusively to tyramine. It is activated by sub-micromolar concentrations of tyramine and mediates Ca^2+^ signals when expressed in Chinese hamster ovary (CHO) cells or *Xenopus* oocytes [65,66]. Similar characteristics, including high selectivity for tyramine and activation of the inositol 1,4,5-trisphosphate/Ca^2+^ signaling pathway, have been shown for the type 2 tyramine receptor of *B. mori* (BmTAR2 [67]). In contrast, the recently characterized type 2 tyramine receptor of *A. mellifera* (AmTAR2) specifically induces cAMP production upon activation [68]. Furthermore, a third tyramine receptor has been characterized in *D. melanogaster* (DmTAR3 [66]). Although DmTAR3 is highly similar to DmTAR2, its activation leads to both a decrease in [cAMP]_i_ and an increase in [Ca^2+^]_i_ [66]. Compared to the other tyramine receptor subtypes, DmTAR3 shows a rather broad ligand spectrum [66].

In *P. americana*, one octopamine receptor (PeaOCTαR1 [69]) and a type 1 tyramine receptor (PeaTAR1A [70]) have been characterized so far among other GPCRs (Pea5-HT_1_ [71]; PeaDOP2 [72]; and PeaGB1/PeaGB2 [73,74]). Here, we report the cloning of a cDNA encoding a second type 1 tyramine receptor from *P. americana* brain (PeaTAR1B). To our knowledge, the existence of two type 1 tyramine receptors have not been shown for any other insect. To investigate the pharmacological properties of PeaTAR1B, a cell line was established that constitutively expresses this GPCR. Activation of the heterologously expressed PeaTAR1B results in the specific inhibition of adenylyl cyclase activity. The receptor’s pharmacological profile was established after applying a concentration series of its agonist tyramine. Receptor activity was efficiently blocked by yohimbine and mianserin. Thus, this study provides new detailed data regarding the pharmacological characteristics of the tyramine receptor system in an important insect pest.

## 2. Results

### 2.1. Cloning of Peatar1b cDNA and Structural Properties of PeaTAR1B

Using degenerate oligonucleotide primers for highly conserved GPCR transmembrane (TM) regions 6 and 7, we amplified a cDNA fragment of 113 bp from *P. americana* brain cDNA coding for a putative tyramine receptor. Rapid amplification of cDNA ends (RACE) was undertaken with gene-specific primers to obtain the missing 5′ and 3′ parts of the putative *Peatar1b* cDNA (see Materials and Methods). The complete cDNA consists of 1706 nucleotides and was independently amplified as a complete fragment by using two gene-specific primers (*Peatar1b*; accession#: LT900530). The longest open reading frame encodes a protein of 481 amino acids (PeaTAR1B) with a predicted molecular mass of 54.1 kDa.

**Figure 1:**
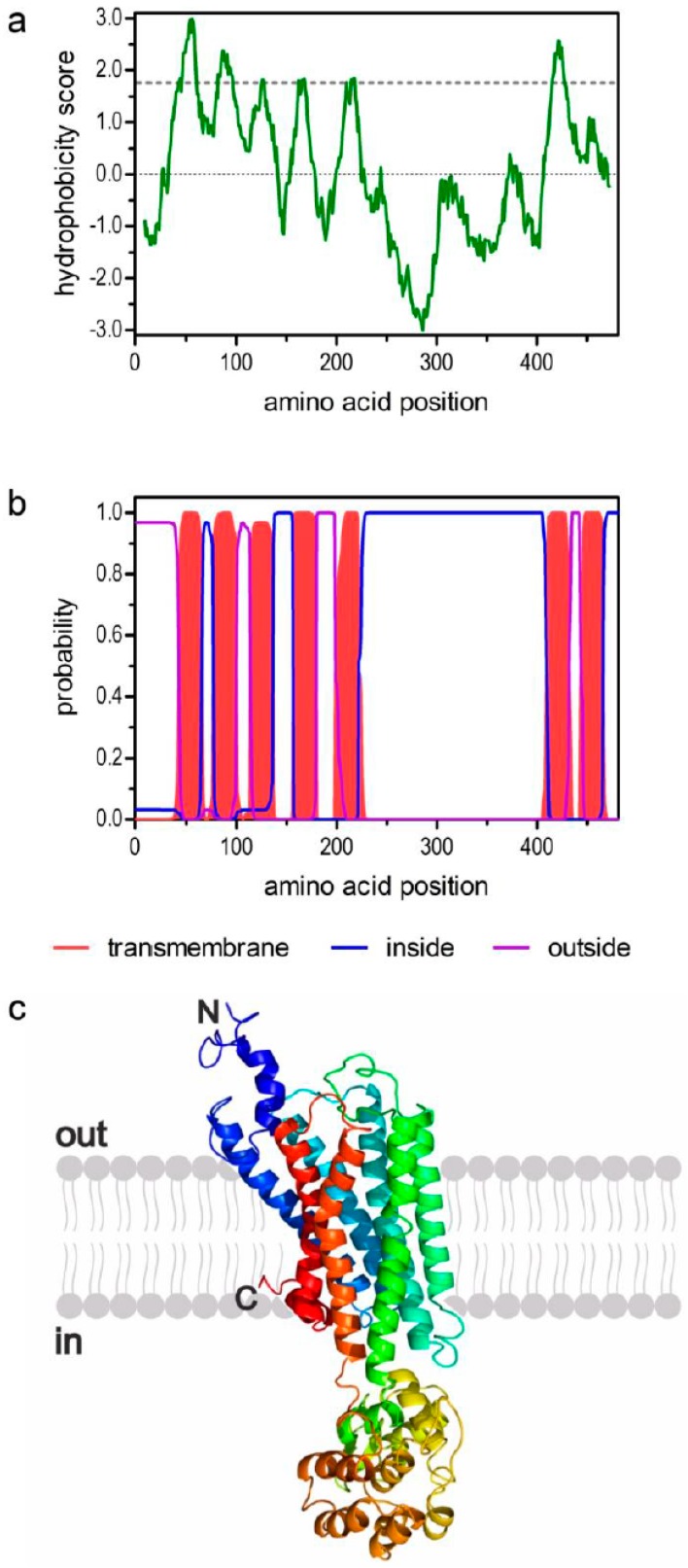
Structural characteristics of the deduced amino acid sequence of PeaTAR1B. (**a**) Hydrophobicity profile of PeaTAR1B. The profile was calculated according to the algorithm of Kyte and Doolittle [75] using a window size of 19 amino acids. Peaks with scores greater than 1.6 (dashed line) indicate possible transmembrane regions; (**b**) Prediction of transmembrane domains with TMHMM server v. 2.0 [76]. Putative transmembrane domains are indicated in red. Extracellular regions are shown as purple line, intracellular regions as blue line; (**c**) Color-coded (rainbow) 3D model of the receptor as predicted by Phyre2 [77]. The extracellular N-terminus (N) and the intracellular C-terminus (C) are labeled.

The hydrophobicity profile according to Kyte and Dolittle [75] and prediction of transmembrane helices using TMHMM Server v. 2.0 [76] suggest seven trans-membrane (TM) domains (Figure 1a,b), which is a characteristic feature of GPCRs. The TM segments are flanked by an extracellular N-terminus of 42 residues and a short intracellular C-terminus of 16 residues. We submitted the PeaTAR1B sequence to Phyre2 [77] and obtained a three-dimensional model of the receptor (Figure 1c).

**Figure 2:**
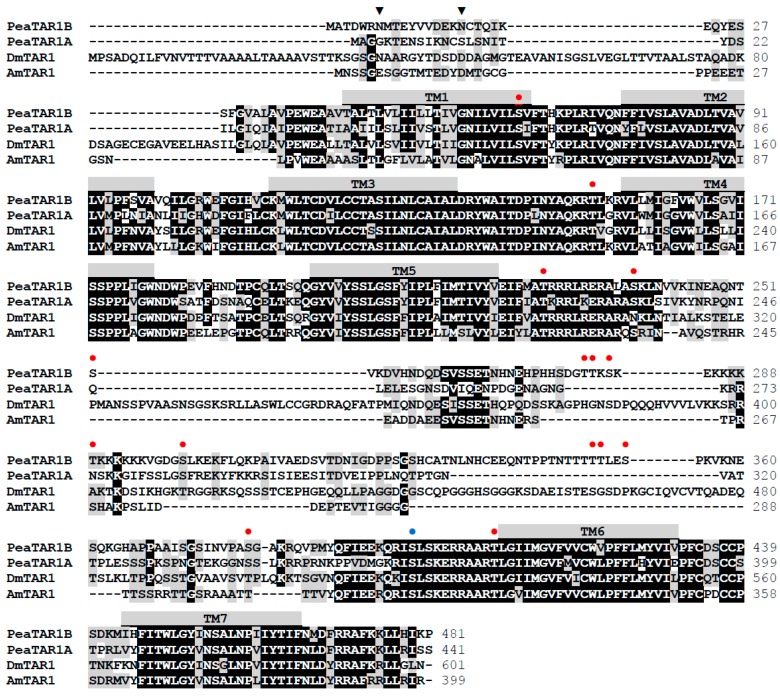
Amino acid sequence alignment of PeaTAR1B and orthologous receptors from *Periplaneta americana* (PeaTAR1A, CAQ48240.1), *Drosophila melanogaster* (DmTYR1; NP_524419.2), and *Apis mellifera* (AmTAR1, NP_001011594.1). Identical residues (≥75%) are shown as white letters against black, whereas conservatively substituted residues are shaded. Putative transmembrane domains (TM1–TM7) are indicated by gray bars. Potential *N*-glycosylation sites (▼), protein kinase A (PKA) phosphorylation sites (●), and protein kinase C (PKC) phosphorylation sites (●) of PeaTAR1B are indicated. The amino acid position is given on the right.

Sequence motifs which are essential for three-dimensional structure, ligand binding, and signal transduction of the receptor are well conserved between the various type 1 tyramine receptors (Figure 2) and are also present in PeaTAR1B. Two consensus motifs for potential *N*-glycosylation (N-X-S/T) are in the extracellular N-terminus of PeaTAR1B (Figure 2). The C-terminus of PeaTAR1B (and other type 1 tyramine receptors) is very short and consists of only 16 amino acid residues (Figure 2). It lacks cysteine residues that could be the target for palmitoylation, a posttranslational modification that frequently occurs in other GPCRs. A consensus motif allowing interaction of the receptor with PSD-95/Discs-large/Zonula occludentes-1 (PDZ) motif-containing anchoring proteins is also missing. One phosphorylation site for protein kinase A (PKA) and 14 phosphorylation sites for protein kinase C (PKC) are present within intracellular domains of PeaTAR1B (Figure 2).

**Figure 3:**
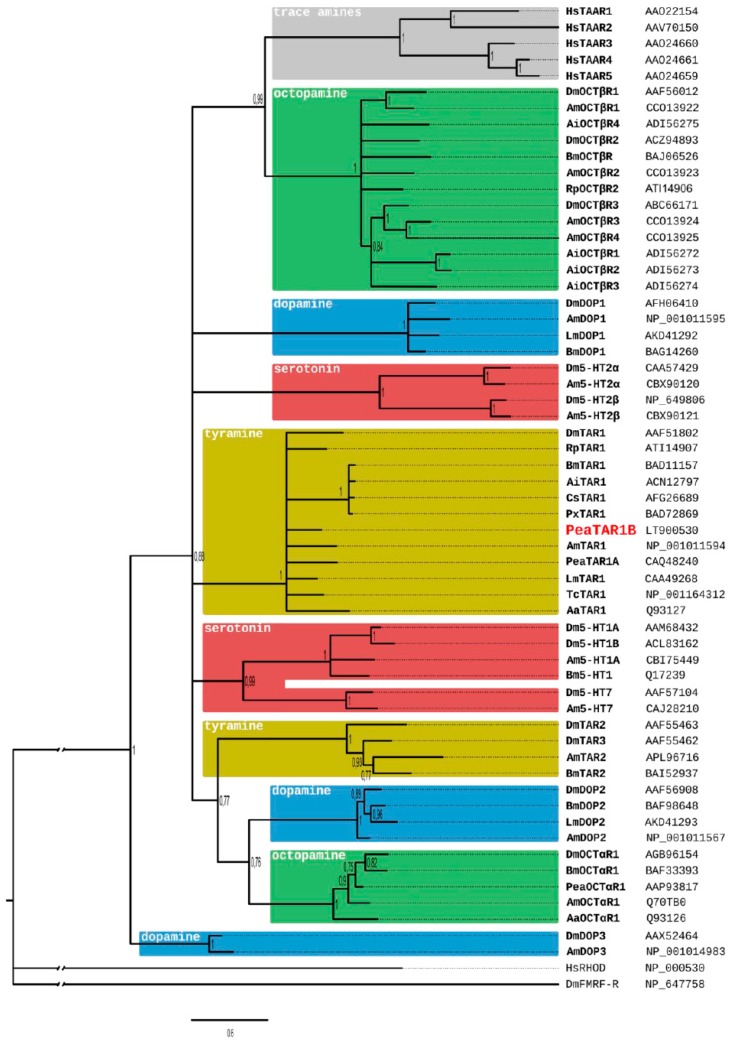
Bayesian phylogeny of insect biogenic amine receptors and trace amine-associated receptors (TAAR). Human rhodopsin (HsRHOD) and fly neuropeptide FMRF amide receptor (DmFMRF-R) were used as outgroup. Numbers at branches represent the posterior probabilities. Nodes with support values below 75% were collapsed. Receptor subclasses are highlighted by distinct colors and the respective ligands are given for each group. Accession numbers are listed behind the receptor’s name. Abbreviations of species in alphabetical order are: Aa, *Amphibalanus amphitrite*; Ai, *Amphibalanus improvisus*; Am, *Apis mellifera*; Bm, *Bombyx mori*; Cs, *Chilo suppressalis*; Dm, *Drosophila melanogaster*; Hs, *Homo sapiens*; Lm, *Locusta migratoria*; Pea, *Periplaneta americana*; Px, *Papilio xuthus*; Rp, *Rhodnius prolixus*; Tc, *Tribolium castaneum*.

A comparison of the PeaTAR1B amino-acid sequence with NCBI databases identified several orthologous type 1 tyramine receptors. The highest amino acid identity (ID) and similarity (S) was found with the RpTAR1 receptor of the blood-sucking bug *Rhodnius prolixus* ([78]; ID 65.6%, S 74.9%) and the LmTAR1 receptor of the migratory locust *Locusta migratoria* ([60]; ID 64.5%, S 74.1%). Homology was also pronounced to tyramine receptors of the lepidopterans *Agrotis ipsolon* ([79]; ID 59.0%, S 72.7%), *Heliothis virescens* ([80]; ID 60.0%, S 72.3%), *Bombyx mori* ([62,63]; ID 58.6%, S 71.3%) and *Papilio xuthus* ([81]; ID 57,0%, S 70.7%), the PeaTAR1A receptor from *P. americana* ([70]; ID 58.4%, S 71.3%), and type 1 tyramine receptors of the honeybee *Apis mellifera* ([61,64]; ID 54.1%, S 65.9%) and *Drosophila melanogaster* ([59]; ID 47.1%, S 59.0%). The phylogenetic relationship of the PeaTAR1B receptor was examined using a Bayesian analysis (Figure 3). For each biogenic amine, various receptor classes exist which are phylogenetically not necessarily closely related. PeaTAR1B assembles in a clade that contains type 1 tyramine receptors from various insect species (Figure 3). In contrast, type 2 tyramine receptors are clearly set apart and form a sister group with invertebrate-type dopamine receptors and adrenergic-like octopamine receptors.

### 2.2. Functional and Pharmacological Properties of PeaTAR1B

**Figure 4:**
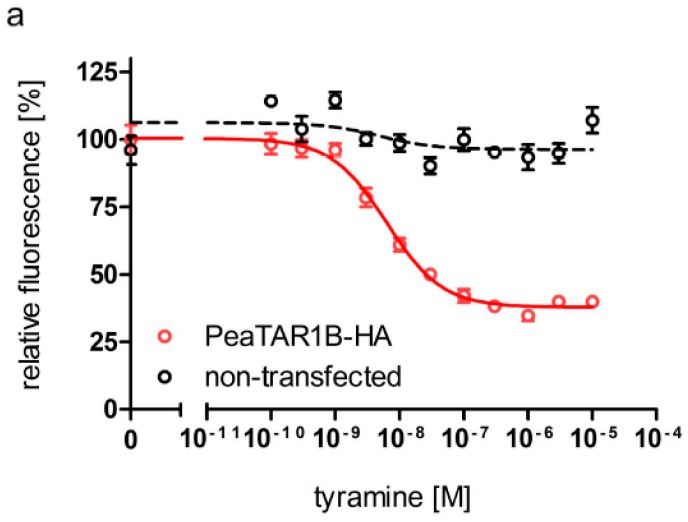

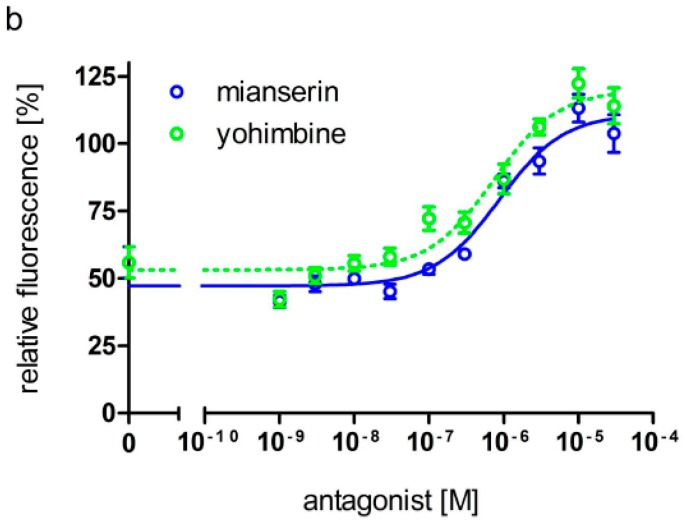
Pharmacological characterization of the PeaTAR1B receptor. (**a**) Concentration-dependent effects of tyramine on intracellular cAMP levels in HEK 293 (flpTM) cells constitutively expressing the PeaTAR1B-HA receptor and in non-transfected cells. Relative fluorescence (corresponding to the amount of cAMP) is given as the percentage of the value obtained with 1 µM NKH 477 (=100%), a water-soluble forskolin analog. All measurements were performed in the presence of 100 µM isobutylmethylxanthine (IBMX). Data points represent the mean ± SD of eight values (PeaTAR1B-HA) or four values (non-transfected) from a typical experiment; (**b**) Effects of putative tyramine receptor antagonists on NKH 477-stimulated cAMP production in PeaTAR1B-HA expressing cells. Concentration series of the substances were applied in the presence of 1 µM NKH 477, 10 nM tyramine and 100 µM IBMX. Data represent the mean ± SD of four values from a typical experiment. Determinations for both (**a**,**b**) were independently repeated at least three times.

For pharmacological characterization of PeaTAR1B, we generated a cell line that constitutively expressed the PeaTAR1B-HA receptor. Due to its close phylogenetic relationship to PeaTAR1A [70], we expected that PeaTAR1B might also attenuate cAMP production in the cell line. To examine PeaTAR1B-HA’s coupling properties, cells were treated with a water-soluble forskolin analog, NKH 477, which stimulates membrane-bound adenylyl cyclases and thereby causes cAMP production in PeaTAR1B-HA expressing cells as well as in non-transfected cells. We examined the concentration-dependent effect of tyramine on NKH 477-stimulated cAMP production. As expected, tyramine attenuated the NKH 477-stimulated production of cAMP in PeaTAR1B-HA expressing but not in non-transfected HEK 293 cells (Figure 4a). Half-maximal reduction of cAMP levels (EC_50_) was observed with ~6.3 nM tyramine (logEC_50_ = −8.20 ± 0.07, mean ± SD; see Figure 4a). The maximal reduction of cAMP synthesis (~60%) was achieved with tyramine concentrations of ≥0.1 µM (Figure 4a). In comparison to PeaTAR1A-HA (EC_50_ = 350 nM), the potency of PeaTAR1B-HA to tyramine is 60-fold higher. In our previous paper [70], we observed that other biogenic amines, e.g., octopamine and dopamine, caused an increase in intracellular cAMP in both PeaTAR1A-HA-expressing and non-transfected cells. The responses even exceeded the values obtained with NKH 477. Since we transfected the same parental cell line with the *Peatar1b*-HA expression construct, we only briefly tested for the effect of other biogenic amines on receptor-expressing and non-transfected cells. The responses were very similar to those described in [70], suggesting that the parental cell line most likely expresses receptors that can be activated by octopamine and dopamine causing either cAMP and/or direct Ca^2+^ responses. Due to these results on the parental cell line, we could not investigate whether octopamine binds to PeaTAR1B-HA. However, all type 1 tyramine receptors examined so far [39,43] cause an inhibition of the enzyme upon tyramine and/or octopamine application. As PeaTAR1B-HA also attenuates cAMP production in response to tyramine, we suggest classifying this protein as a tyramine rather than a tyramine/octopamine receptor.

Putative antagonists that previously have been shown to compete for tyramine-dependent attenuation of cAMP synthesis were also tested. The effect of tyramine (10 nM) could be blocked by co-incubation with mianserin and the indole alkaloid yohimbine in a concentration dependent manner (Figure 4b). The calculated IC_50_ values for mianserin and yohimbine were 8.68 × 10^−7^ M (logEC_50_ = −6.06 ± 0.11, mean ± SD) and 7.40 × 10^−7^ M (logEC_50_ = −6.13 ± 0.12, mean ± SD), respectively. None of chlorpromazine, cyproheptadine, epinastine and phentolamine could inhibit the tyramine-induced reduction in cAMP synthesis.

## 3. Discussion

There is ongoing interest in precisely understanding the physiological and behavioral roles of tyraminergic/octopaminergic signaling in insects [42,43,44,82,83,84,85]. To meet this challenge, important steps are to determine the molecular and functional-pharmacological properties of tyramine and octopamine receptor subtypes and to address their tissue and cellular distribution, especially within the central nervous system (CNS). Based on a rich body of data, a picture emerges that the tyramine/octopamine system of protostomes functionally substitutes the norepinephrine/epinephrine system of deuterostomes [86]. Therefore, tyramine and octopamine receptors may represent interesting targets for relatively selective insecticides with low toxicity for vertebrates. Using model insects such as *P. americana* might accelerate the gain of knowledge. Here, we have focused on elucidating the pharmacological properties of a second type 1 tyramine receptor from *P. americana*, PeaTAR1B. A cell line constitutively expressing PeaTAR1B can now be used for high-throughput screening of potential agonists and antagonists in search for effective and selective insecticides to fight this and other insect pests. Recently, such a strategy has proven very successful for an α-adrenergic-like octopamine receptor of the malaria vector *Anopheles gambiae* [87].

### 3.1. Molecular Features of the PeaTAR1B Receptor

Most class A (or rhodopsin-like) GPCRs are activated via agonists docking to specific residues in the binding pocket of the receptor near the extracellular side. Functionally important amino acid residues present in type 1 tyramine receptors [63] are well conserved in the PeaTAR1B sequence. These are an aspartic acid residue (D^3.32^, nomenclature according to [88]; D_120_ in PeaTAR1B) in TM3 and two of three closely grouped serine residues found in TM5 (S^5.42, 5.46^; S_204, 208_) (see Figure 2). Tyramine appears to bind via its amine group and its hydroxyl group to the aspartic acid and one of the serine residues of the receptor, respectively [63,89]. In addition, phenylalanine and/or tryptophan residues in TM6 and TM7 (see Figure 2) might contribute to π–π interaction with delocalized electrons in tyramine and stabilize the receptor ligand interaction.

The coupling of GPCRs to specific G proteins is brought about by amino-acid residues in close vicinity to the plasma membrane of the 2nd and 3rd intracellular loops and of the cytoplasmic C-terminus of the receptor. Biogenic amine receptors that couple to G_i_ proteins and thereby inhibiting adenylyl cyclase activity often possess short C termini [90]. This feature is conserved in PeaTAR1B and in other type 1 tyramine receptors (Figure 2). In addition, the receptors possess strikingly similar amino-acid sequences in the vicinity of TM5 and TM6 within their 3rd cytoplasmic loops, a region largely determining the specificity of receptor/G-protein coupling [91,92].

### 3.2. Pharmacological Properties of PeaTAR1B

The PeaTAR1B receptor was functionally expressed in HEK 293 cells. Coupling of PeaTAR1B to intracellular signaling cascades was examined via cell-endogenous G-proteins. PeaTAR1B, like other type 1 tyramine receptors from various insects [33,55,59,60,61,62,63,64,68,70] and the nematode *C. elegans* [93,94], is negatively coupled to the enzyme adenylyl cyclase via G_i_ proteins, and thus results in a decrease in [cAMP]_i_. With an EC_50_ of 6.3 nM, activation of PeaTAR1B was much more sensitive to tyramine than the PeaTAR1A receptor (EC_50_ = 350 nM) which was recently characterized using the same heterologous expression system [70]. The data obtained for heterologously expressed receptors agree well with those from various native tissue preparations. Tyramine has been shown to reduce adenylyl cyclase activity in *D. melanogaster* head homogenates [95], membrane fractions of *A. mellifera* brains [61], and head membrane preparations of *B. mori* [96,97].

Inhibition of receptor-mediated attenuation in [cAMP]_i_ in the cell line constitutively expressing PeaTAR1B was examined with various synthetic antagonists. In addition to yohimbine (IC_50_ = 7.40 × 10^−7^ M) which is also an antagonist of PeaTAR1A [70] and most other type 1 tyramine receptors [33,59,60,62,68], the action of tyramine on PeaTAR1B could also be blocked by mianserin (IC_50_ = 8.68 × 10^−7^ M) with a similar efficacy. Mianserin is primarily known as antagonist/inverse agonist at 5-HT_2_ serotonin receptors in mammals, where it also blocks H_1_ histamine receptors and α_2_ adrenoceptors. In insects, however, besides blocking various 5-HT receptors (e.g., [98,99,100,101]), mianserin is known as a potent antagonist at octopamine receptors [102,103]. More recently, mianserin was found to be an antagonist of the AmTAR2 receptor of the honeybee [68]. Chlorpromazine, an anti-psychotic drug that blocks mammalian D_2_ dopamine and H_1_ histamine receptors, has been shown to inhibit, e.g., PeaTAR1A [70] and honeybee tyramine receptors [68]. The activity of tyramine-stimulated PeaTAR1B, however, was not affected by chlorpromazine. Other substances, i.e., cyproheptadine, epinastine and phentolamine, also completely lacked inhibitory potential on the PeaTAR1B receptor.

### 3.3. Expression Pattern

Clues to the physiological significance and/or possible function of a GPCR might be obtained from its cellular localization. Although we have not experimentally addressed the expression pattern of the *Peatar1b* gene in this study, we had the opportunity to screen transcriptome data from various tissues of the cockroach (R. Predel, University of Cologne, personal communication). Both the *Peatar1a* mRNA and the *Peatar1b* mRNA was detected in transcriptomes of the brain (frontal ganglion) and the glandular part of the *corpora cardiaca*. In addition, *Peatar1a* mRNA but not *Peatar1b* mRNA was detected in transcriptomes of the heart and muscles. Neither *Peatar1a* mRNA nor *Peatar1b* mRNA could be detected in transcriptomes of the midgut. In an earlier study, we successfully amplified *Peatar1a* transcripts by RT-PCR in RNA samples prepared from brains, salivary glands, Malpighian tubules, leg muscles, and midgut of adult cockroaches, with the lowest amount detected in midgut samples [70].

In conclusion, the occurrence of two functional type 1 tyramine receptors with different pharmacological profiles in *P. americana* is an important new finding and has not yet been described for any other species. Although speculative at this point, one might consider assembly of PeaTAR1A/PeaTAR1B heteromers displaying pharmacological profiles that deviate from homomeric receptors. However, independent of their oligomeric structures, these receptors converge on physiologically reducing intracellular [cAMP]_i_. Whether *P. americana* expresses type 2 tyramine receptors has to be addressed in future studies.

## 4. Materials and Methods

### 4.1. Cloning of the Peatar1b cDNA

Based on sequence conservation throughout various arthropod species, degenerate primers (DF1: 5′-TGYTGGBTICCITTYTT-3′; DR1: 5′-CCARCAISHRTADATIAYIGGRTT-3′) were designed to amplify cDNA fragments of *P. americana* aminergic receptors [2,70,71,72]). Polymerase chain reaction (PCR) was performed on a *P. americana*-brain cDNA library [104] under the following conditions: 1 cycle of 2.5 min at 94 °C, followed by 35 cycles of 40 s at 94 °C, 40 s at 45 °C, and 20 s at 72 °C, and a final extension of 10 min at 72 °C. The PCR products were cloned into pGEM-T vector (Promega, Mannheim, Germany) and subsequently analyzed by DNA sequencing (AGOWA, Berlin, Germany). Missing 5′- and 3′-regions of the cDNA were amplified by SMART RACE (rapid amplification of cDNA ends) experiments (Clontech, Saint-Germain-en-Laye, France). Finally, a PCR was performed on single-stranded *P. americana*-brain cDNA to amplify the entire coding region of *Peatar1b* by using gene-specific primers annealing in the 5′- and 3′-untranslated regions (SF: 5′-CATCGTGTGGTATTTCACTCATTC-3′; SR: 5′-GGACCACAGTGAATATGAACCC-3′). The nucleotide sequence of *Peatar1b* has been submitted to the European Bioinformatics Institute (EMBL-EBI) database (accession#: LT900530). *N*-glycosylation sites were predicted by NetNGlyc 1.0 Server (http://www.cbs.dtu.dk/services/NetNGlyc/). Putative phosphorylation sites were predicted by NetPhos 3.1 Server (http://www.cbs.dtu.dk/services/NetPhos/; [105]).

### 4.2. Multiple Sequence Alignments and Phylogenetic Analysis

Amino acid sequences used for phylogenetic analyses were identified by protein-protein BLAST searches of the NCBI database with the deduced amino acid sequence of *Peatar1b* (PeaTAR1B) as “bait”. Multiple sequence alignments of the complete amino acid sequences were performed with ClustalW and trimmed to the regions from TM1–5 and TM6–7. Afterwards, a Bayesian analysis (MrBayes v3.2.6; [106]) was performed with 1,000,000 generations, an initial burn in of 2500, and the substation model LG + I + G, determined by Protest 3.4.2 [107]. Nodes with support values below 75% were collapsed.

Values for identity (ID) and similarity (S) of type 1 tyramine receptors were calculated by using the BLOSUM62 substitution matrix in BioEdit 7.2.6 [108] after pairwise alignment.

### 4.3. Construction of Expression Vectors

An expression-ready construct of *Peatar1b* in pcDNA3.1(+) vector was generated by PCR. Specifically, receptor encoding cDNA was modified in a PCR with primers *Peatar1b*-expr-F (5′-TTTAAGCTTCCACCATGGCGACTGACTGGAGAAATATG-3′) and *Peatar1b*-expr-R (5′-TTTGAATTCTGGTTTGATGTGGAGTAATTTTTTG-3′). In front of the start codon, a Hind*III* restriction site (AAGCTT) and a Kozak consensus motif (CCACC; [109]) were inserted. The stop codon was replaced by an Eco*RI* recognition sequence (GAATTC). We reused the pc*Am5-ht1A*-HA construct [110] and exchanged the *Am5-ht1A* cDNA for the *Peatar1b* cDNA by ligation into the Hind*III* and Eco*RI* sites. The resulting construct (pc*Peatar1b*-HA) is extended in frame at the 3′ end with a sequence encoding the hemagglutinin A (HA) tag (YPYDVPDYA) which allowed us to monitor receptor protein expression using a specific anti-HA antibody (Roche Applied Science, Mannheim, Germany). The correct insertion was confirmed by DNA sequencing.

### 4.4. Functional Expression in Mammalian Cell Lines

For PeaTAR1B-HA expression and pharmacological analysis, we used a cell line that had been transfected with a gene encoding a variant of the A2-subunit of the olfactory cyclic nucleotide-gated ion channel (CNG; [111]; flpTM cells, provided by Sibion biosciences, Jülich, Germany). These flpTM cells were transfected with 10 µg of the pc*Peatar1b*-HA construct by a modified calcium phosphate method [112] following a previously established protocol [68,73,99,113]. Transfected cells were selected in the presence of the antibiotics G418 (1 mg/mL) and hygromycin (100 µg/mL). Expression of PeaTAR1B-HA receptor was monitored by Western blotting and immunocytochemistry using anti-HA antibodies (Roche Applied Science).

### 4.5. Functional Characterization of the PeaTAR1B Receptor

A cell line expressing the highest amount of the receptor was used to examine PeaTAR1B receptor activity by Ca^2+^ imaging. Control measurements were performed in the parental (flpTM) cell line. Changes in intracellular cAMP concentrations were registered indirectly via the co-expressed CNG channels that are opened by cAMP and cause an influx of extracellular Ca^2+^ [68,73,99,113]. Changes in [Ca^2+^]_i_ were monitored with the Ca^2+^-sensitive fluorescent dye Fluo-4. Cells were grown in 96-well dishes to a density of approximately 2 × 10^4^ cells per well and were loaded at room temperature with Fluo-4 AM as described previously [68,73,99]. After 90 min, the loading solution was substituted for dye-free extracellular solution (ECS; 120 mM NaCl, 5 mM KCl, 2 mM MgCl_2_, 2 mM CaCl_2_, 10 mM 2-[4-(2-hydroxyethyl)piperazin-1-yl]ethanesulfonic acid (HEPES), and 10 mM glucose, pH 7.4 (NaOH)) containing 100 µM IBMX. Measurements on cells expressing PeaTAR1B-HA were performed in the presence of 1 µM NKH477 (activator of membrane-bound adenylyl cyclases; NKH 477 was from Tocris—Biotrend, Cologne, Germany), since PeaTAR1B-HA was predicted to inhibit adenylyl cyclase activity. The plate was transferred into a fluorescence reader (FLUOstar Omega, BMG Labtech, Offenburg, Germany) to monitor Fluo-4 fluorescence. The excitation wavelength was 485 nm. Fluorescence emission was detected at 520 nm. Concentration series of various biogenic amines and synthetic receptor ligands were added once Fluo-4 fluorescence had reached a stable value in each well. Receptor ligands (biogenic amines: dopamine, histamine, octopamine, serotonin, and tyramine; and potential antagonists: chlorpromazine, cyproheptadine, epinastine, mianserin, phentolamine, and yohimbine) were all purchased from Sigma (Taufkirchen, Germany). The changes in Fluo-4 fluorescence were recorded automatically. Concentration–response curves were established from at least three independent experiments with quadruplicate or octuplicate measurements. Data were analyzed and displayed using Prism 5.04 software (GraphPad, San Diego, CA, USA).

## 5. Conclusions

The cockroach *P. americana* contains two genes which both code for type 1 tyramine receptors, PeaTAR1A [70] and PeaTAR1B (this study). The proteins share 71.3% of similarity. When heterologously expressed in a eukaryotic cell line, both receptors attenuate NKH 477-stimulated cAMP production upon tyramine application in a concentration dependent manner. The PeaTAR1B receptor is approximately 60-fold more sensitive to the biogenic amine (EC_50_ 6.3 nM) compared to PeaTAR1A (EC_50_ 350 nM). Pharmacologically PeaTAR1B was efficiently blocked by mianserin and yohimbine, whereas other potential receptor antagonists were non-effective. Whether PeaTAR1A and PeaTAR1B represent the complete inventory of tyramine receptors in this insect remains to be established.

## Abbreviations

cAMP	cyclic adenosine monophosphate (3′,5′-cyclic adenosine monophosphate)
GPCR	G protein-coupled receptor
IBMX	3-isobutyl-1-methylxanthine
ID	identity
NCBI	National Center for Biotechnology Information
PKA	protein kinase A (AMP-dependent protein kinase)
PKC	protein kinase C
S	similarity
TAAR	trace amine-associated receptor
TM	transmembrane domain
